# Predicting tumor recurrence on baseline MR imaging in patients with early-stage hepatocellular carcinoma using deep machine learning

**DOI:** 10.1038/s41598-023-34439-7

**Published:** 2023-05-10

**Authors:** Ahmet Said Kucukkaya, Tal Zeevi, Nathan Xianming Chai, Rajiv Raju, Stefan Philipp Haider, Mohamed Elbanan, Alexandra Petukhova-Greenstein, MingDe Lin, John Onofrey, Michal Nowak, Kirsten Cooper, Elizabeth Thomas, Jessica Santana, Bernhard Gebauer, David Mulligan, Lawrence Staib, Ramesh Batra, Julius Chapiro

**Affiliations:** 1 Department of Radiology and Biomedical Imaging, Yale University School of Medicine, 330 Cedar Street, New Haven, CT 06520-8042 USA; 2 Institute of Radiology, Charité-Universitätsmedizin Berlin, Corporate Member of Freie Universität Berlin, Humboldt-Universität, and Berlin Institute of Health, Augustenburger Platz 1, 13353 Berlin, Germany; 3 Department of Diagnostic Radiology, Bridgeport Hospital, Yale New Haven Health System, 267 Grant Street, Bridgeport, CT 06610 USA; 4 Visage Imaging, Inc., 12625 High Bluff Drive, Suite 205, San Diego, CA 92130 USA; 5 Transplantation and Immunology, Department of Surgery, Yale University School of Medicine, 333 Cedar Street, New Haven, CT 06520 USA

**Keywords:** Medical research, Hepatocellular carcinoma, Predictive markers, Magnetic resonance imaging, Machine learning

## Abstract

Tumor recurrence affects up to 70% of early-stage hepatocellular carcinoma (HCC) patients, depending on treatment option. Deep learning algorithms allow in-depth exploration of imaging data to discover imaging features that may be predictive of recurrence. This study explored the use of convolutional neural networks (CNN) to predict HCC recurrence in patients with early-stage HCC from pre-treatment magnetic resonance (MR) images. This retrospective study included 120 patients with early-stage HCC. Pre-treatment MR images were fed into a machine learning pipeline (VGG16 and XGBoost) to predict recurrence within six different time frames (range 1–6 years). Model performance was evaluated with the area under the receiver operating characteristic curves (AUC–ROC). After prediction, the model’s clinical relevance was evaluated using Kaplan–Meier analysis with recurrence-free survival (RFS) as the endpoint. Of 120 patients, 44 had disease recurrence after therapy. Six different models performed with AUC values between 0.71 to 0.85. In Kaplan–Meier analysis, five of six models obtained statistical significance when predicting RFS (log-rank p < 0.05). Our proof-of-concept study indicates that deep learning algorithms can be utilized to predict early-stage HCC recurrence. Successful identification of high-risk recurrence candidates may help optimize follow-up imaging and improve long-term outcomes post-treatment.

## Introduction

Hepatocellular carcinoma (HCC) is the second most common cause of cancer-related death worldwide^1,2^. Although early-stage HCC patients are treated with curative intent, the recurrence rates remain high. Recurrence risk has been noted to be as high as 70% for patients treated with surgical resection^3^ or thermal ablation^4^ and about 20% with orthotopic liver transplantation (OLT)^5^ within 5 years post-treatment. Although patients with recurrence may undergo image-guided locoregional therapy or adjuvant systemic treatment with targeted agents such as sorafenib, supporting evidence for these approaches is limited^6–8^.

The diagnosis and staging of HCC are mainly based on cross-sectional body imaging, such as magnetic resonance (MR) imaging and computer tomography (CT). The size and number of tumors are the main criteria forming the foundation of various staging systems, for instance, the Barcelona Clinic Liver Cancer (BCLC) system^9^. But, the lesion number and size do not adequately characterize the tumor biology or its characteristic imaging appearance. Thus, these systems cannot accurately estimate the patient’s post-treatment recurrence risk.

Some recent studies have studied the value of machine learning algorithms to predict HCC recurrence^10–13^. So far, these studies have used a predefined catalog of manually hand-crafted features to predict HCC recurrence^10,12,14^. This approach has some drawbacks, since manually hand-crafted features may not capture cancer’s pathophysiological properties entirely. That means, they are created by humans and may be subject to selection bias. In contrast, deep learning algorithms can automatically extract the most relevant and predictive imaging features, without human engineering. Deep learning algorithms can also be utilized in combination with medical imaging data, to support a wide range of applications, including automated segmentation, lesion classification, and abnormality detection^15^. Its hallmark lies in the advanced ability to deal with unstructured data, such as medical images^16^, and to automatically extract relevant imaging features that may not be apparent to even the most experienced human eyes^17^.

Even though there are already studies on HCC recurrence prediction utilizing deep learning algorithms, most of these studies use CT imaging as input^18^. In contrast, we ask whether using MR imaging instead of CT is more promising due to the higher soft tissue contrast of MR imaging compared to CT. To the best of our knowledge, there is no study utilizing deep learning for HCC recurrence prediction in combination with MR imaging for early-stage HCC.

This study aims to build, validate, and test a deep learning approach to predict the post-treatment (surgical, ablative, or OLT) recurrence risk in early-stage HCC patients from pre-treatment MR imaging.

## Methods

### Patient population

This was a single-center retrospective study compliant with the Health Insurance Portability and Accountability Act (HIPAA). This study was approved by the institutional review board of Yale University and informed consent was waived. The study was performed in accordance with the Declaration of Helsinki. One hundred and twenty adults (≥ 18 years) from 2005 to 2018 were included. From a total of 1190 patients treated from January 2005 to December 2018 for HCC at our institution, 120 patients were included according to the following criteria: (1) either histopathologically or imaging-criteria confirmed HCC, (2) either stage 0 or stage A, according to the most recent BCLC staging system^19^, (3) surgical resection, thermal ablation, or OLT as first-line, stand-alone treatment after initial listing/consideration for transplant in all three scenarios, (4) available multi-parametric contrast-enhanced pre-treatment MR imaging, and (5) complete response after treatment, defined as the disappearance of all signs of viable tumor at first post-interventional follow-up imaging. Exclusion criteria were as follows: (1) imaging confirmed macrovascular invasion or metastatic HCC, (2) presence of active co-malignancies from the time of HCC diagnosis through the entire follow-up period, and (3) excessive motion artifact on pre-treatment MR imaging. Pre-treatment clinical variables were collected. Patient data were extracted from the Electronic Health Records. Further information regarding patient characteristics can be found in Table 1.

**Table 1:** Baseline patient characteristics.

Characteristic	Overall (n = 120)	Recurrence (n = 44)	No-recurrence (n = 76)	*p* *α* = 0.00185
Age, median (IQR)	60 (11)	60 (14.3)	59 (10)	
BMI, median (IQR)	27.7 (7.6)	27.3 (5.8)	28.3 (7.9)	0.221
Gender, N (%)				0.753
Male	88 (73.3)	33 (75)	55 (72.4)	
Female	32 (26.7)	11 (25)	21 (27.6)	
Etiology, N (%)				0.767
HCV	61 (50.8)	23 (52.3)	38 (50)	
EtOH	11 (9.2)	4 (9)	7 (9.2)	
HBV	5 (4.2)	1 (2.3)	4 (5.3)	
NASH	14 (11.7)	4 (9)	10 (13.2)	
HCV/EtOH	13 (10.8)	6 (13.7)	7 (9.2)	
HCV/EtOH/HBV	1 (0.8)	1 (2.3)	0 (0)	
Others	15 (12.5)	5 (11.4)	10 (13.1)	
Cirrhosis, N (%)	102 (85)	34 (77.2)	68 (89.5)	0.071
Child–Pugh Score, N (%)				0.003
A	82 (68.3)	38 (86.3)	44 (57.9)	
B	29 (24.2)	6 (13.7)	23 (30.3)	
C	9 (7.5)	0 (0)	9 (11.8)	
BCLC stage, N (%)				0.585
0	36 (30)	15 (34.1)	21 (27.6)	
A	83 (69.2)	29 (65.9)	54 (71.1)	
B	1 (0.8)	0 (0)	1 (1.3)	
MELD-Na, median (IQR)	9 (5)	8 (3)	10 (6)	0.004
Portal hypertension, N (%)	62 (51.7)	15 (34.1)	47 (61.8)	0.003
Number of tumors, N (%)				0.402
1	102 (85)	38 (86.4)	64 (84.2)	
2	15 (12.5)	6 (13.6)	9 (11.8)	
3	3 (2.5)	0 (0)	3 (4)	
Max. tumor diameter [cm], median (IQR)	2.3 (1.2)	2.6 (1.4)	2.2 (1.1)	0.083
Cum. tumor diameter [cm], median (IQR)	2.4 (1.6)	2.6 (2.2)	2.4 (1.5)	0.159
Laboratory, median (IQR)				
Serum total bilirubin [mg/dL]	0.9 (1.3)	0.8 (0.9)	1.2 (1.4)	0.004
Serum albumin [g/dL]	3.8 (0.8)	3.9 (0.7)	3.7 (0.9)	0.014
Serum ALT [U/I]	47 (46)	52 (38)	38 (41)	0.026
Serum AST [U/I]	56 (47.8)	58 (43)	56 (56)	0.337
Creatinine [mg/dL]	0.9 (0.4)	0.9 (0.3)	0.9 (0.4)	0.356
INR	1.1 (0.2)	1.1 (0.2)	1.1 (0.3)	0.005
AFP [ng/mL]	9 (26.5)	9 (42)	11 (19)	0.488
NLR	2.2 (1.7)	2.1 (1.7)	2.3 (2.1)	0.074
PLR	4.2 (3.7)	5 (4.8)	3.8 (3.5)	0.028

*BMI* body mass index, *HCV* chronic hepatitis C, *EtOH* alcoholic liver disease, *HBV* chronic hepatitis B, *NASH* non-alcoholic steatohepatitis, *BCLC* Barcelona clinic liver cancer, *MELD* model for end-stage liver disease, *ALT* alanine aminotransferase, *AST* aspartate aminotransferase, *AP* alkaline phosphatase, *GFR* glomerular filtration rate, *INR* international normalized ratio, *AFP* alpha-fetoprotein, *NLR* neutrophil-to-lymphocyte ratio, *PLR* platelet-to-lymphocyte ratio.

Chi-square test was used for categorical and ordinal variables.

Mann–Whitney *U* test was used for continuous variables.

Significance level α is Bonferroni-corrected.

To ensure all patients were diagnosed according to current imaging diagnostic criteria, all MR images were read by three board-certified radiologists (two of them with 7, and one of them with 8 years of experience in body MR Imaging, respectively) according to the most recent Liver Reporting and Data Systems (LI-RADS v2018)^20^ criteria. The dataset was split into three portions. Each radiologist read a portion in independent reads. Patients with at least one LI-RADS (LR)-5 lesion were considered diagnosed with HCC. For patients not meeting LR-5 imaging criteria, biopsy or postoperative pathological confirmation of HCC was considered sufficient for diagnosis.

### Clinical endpoint

Recurrence was defined as the intra- or extrahepatic appearance of HCC after treatment, confirmed by multi-parametric contrast-enhanced imaging or histology. Recurrence included intrahepatic local (within the liver, at the place of the original tumor), intrahepatic distant (within the liver, distant from the original tumor), and extrahepatic (outside the liver) recurrence. Recurrence-free survival (RFS) was defined as the time interval between curative treatment to first imaging evidence of recurrence. Multi-parametric contrast-enhanced MR imaging or computer tomography (CT) of the abdomen was used for follow-up imaging, including pre-contrast, arterial, portal venous, and delayed phase imaging. Patients received imaging every 3 months in the first post-procedure year and every 6 months after that. To monitor for extrahepatic recurrence, patients received a non-contrast chest CT every 6 months.

### Imaging acquisition

Acquisitions from multiple MR imaging scanners with different contrast agents were used to allow for a more robust and generalizable machine learning model. Scanners were 1.5 T and 3 T models produced by Siemens (Aera, Avanto, Espree, TrioTim, Skyra, Verio), General Electric (SIGNA HDx), and Philips (Achieva). Studies were performed with several contrast agents, including Gadavist (Bayer), Magnevist (Bayer), Eovist (Bayer), Multihance (Bracco Diagnostics), Prohance (Bracco Diagnostics), Optimark (Covidien), and Dotarem (Guerbet). Contrast agents were administered at a dose of 1–1.5 mmol/kg with an injection speed of 2–5 mL/s. The input for our model consisted of multi-parametric, gadolinium-enhanced T1-weighted gradient echo acquisitions including arterial phase (35 s post-injection), portal venous phase (70 s post-injection), and delayed phase (3–5 min post-injection) imaging. Repetition and echo times ranged from 2.5–5.5 ms and 1–3 ms, respectively. Pixel bandwidth ranged from 250 to 650 Hz, pixel spacing from 0.5 to 1.7 mm, slice thickness from 3 to 5 mm, number of slices from 43 to 125, and image matrices from 160 × 160 to 415 × 200.

### Machine learning pipeline

#### Image pre-processing

A rectangular, three-dimensional bounding box was placed around the liver in the pre-treatment, multi-parametric MR axial images using 3D Slicer (Version 4.10.2)^21^. The task was performed by a third-year radiology resident, supervised by board-certified subspecialty-trained readers with 7 and 8 years of experience, as detailed above. The whole liver was chosen as the region of interest to capture most of the potentially predictive imaging features. The bounding box was created based on the arterial phase and then adapted to the portal venous and delayed phase images. An exemplary representation of a bounding box can be found in Supplementary Fig. 2. After that, corresponding slices of all three phases were resized to 224 × 224 pixels and combined into one stack. The resizing steps were performed to create a consistent input matrix size of 224 × 224 × 3 pixels required for feature extraction.

#### Feature extraction

Feature extraction and voxel intensity normalization were performed using VGG16^22^. This convolutional neural network (CNN) was pre-trained on a subset of the ImageNet database. The original pre-trained weights were used for feature extraction without fine-tuning the neural network. It was chosen due to its robust performance in various domains^23^, and it is an effective method when only a limited amount of imaging data is available. The voxel intensities were normalized using z-score normalization, as per given the formulae: x_n_ʹ = (x_n_ − µ)/σ, where x_n_ is the original voxel intensity value, µ the mean of all voxel intensity values per patient, σ the standard deviation of all voxel intensity values per patient, and x_n_ʹ the normalized voxel intensity value. In total, 4096 features per stack were extracted from the 2nd fully connected layer of the CNN. Finally, the maximum value per feature across all MR image stacks was pooled to obtain a single feature vector of 4096 features per patient. A more detailed overview of the pre-processing and feature extraction can be found in Supplementary Fig. 1.

#### Recurrence prediction

**Figure 1:**
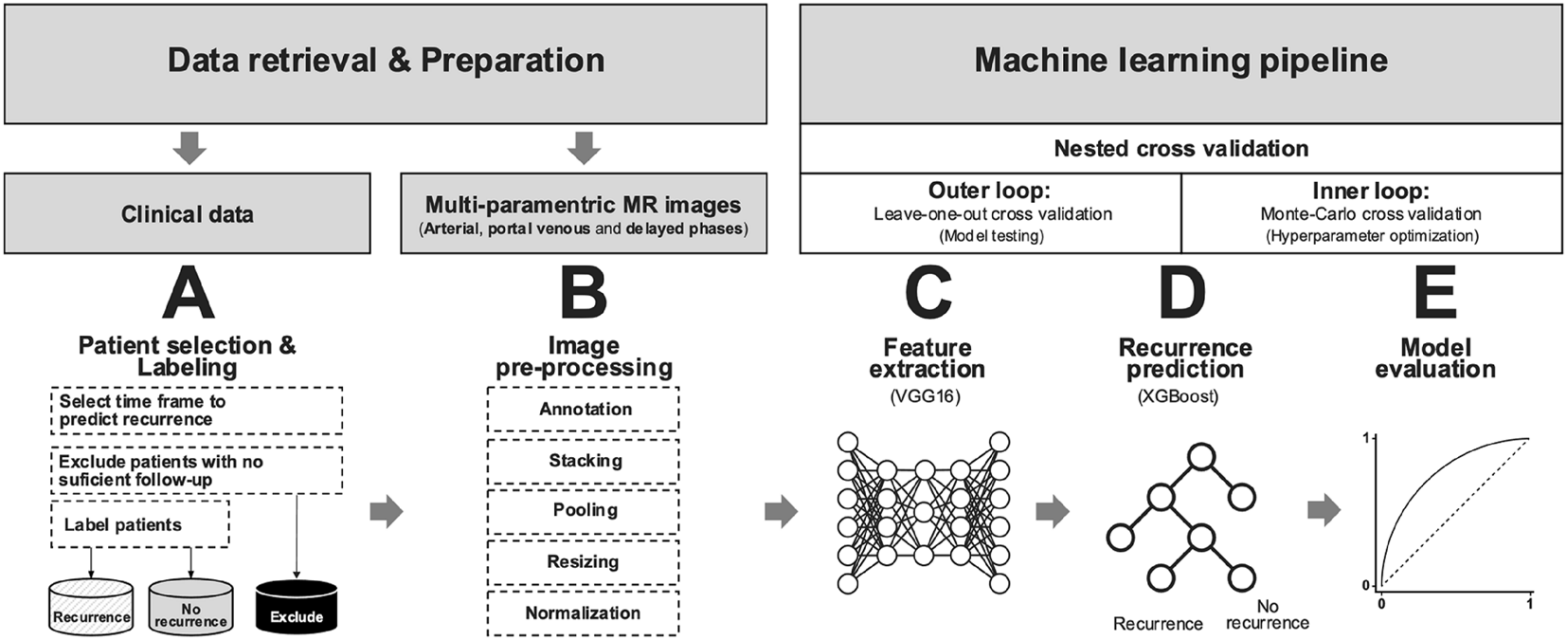
Overview of the machine learning pipeline. (**A**) For each investigated time frame, patients with sufficient follow-up were included and labeled. (**B**) Multiparametric MR images were annotated with three-dimensional bounding boxes to the size of the liver. (**C**) Subsequent pre-processing steps including pooling, pixel intensity normalization, resizing, and stacking were applied before feeding the images to the algorithm. (**D**) VGG16, a CNN trained on 14 million images, was used for feature extraction. Images were converted into a 1 × 4096 feature vector. (**E**) Finally, feature vectors were used to make the final classification, whether a patient recurred or not with XGBoost.

The final imaging feature vector was fed into XGBoost^24^, a state-of-the-art tree-based ensemble classifier. To validate the algorithm, nested cross-validation (CV)^25^ was performed, consisting of an outer- and an inner loop. In the outer loop, leave-one-out CV was used to evaluate the model performance, meaning that all patients but one were used to train, and the remaining one was used to validate the model. In the inner loop, tenfold stratified Monte Carlo CV was used to optimize the model hyperparameters. For this, patients were randomly assigned to training (90%) and validation (10%) sets over six iterations. Figure 1 depicts an overview of our machine learning workflow.

### Outcome endpoint

The Kaplan–Meier method was used to investigate our machine learning pipeline’s clinical relevance and efficiency, with RFS as the outcome of interest. Patients were stratified by the predictions of the models into “high-risk” and “low-risk” recurrence. As the machine learning model only provides a recurrence probability, a specific threshold had to be chosen, against which the patients are stratified. For each time frame, the threshold was selected by dividing the number of all recurrent by the number of all cases. Patients who recurred during the analyzed time frame were marked as events, whereas patients who remained cancer-free were censored. Survival curves were compared using the log-rank test. A p-value below 0.05 was considered significant.

### Statistical analysis

The median and interquartile ranges were used to summarize patient characteristics. Recurrent and non-recurrent patients were compared using the Chi-square test (for categorical and ordinal variables) and the Mann–Whitney *U* test (for continuous variables). Bonferroni correction was used to adjust the significance level for the family-wise error rate in the baseline characteristics. The area under the receiver operating characteristic curve (AUC–ROC) was used to evaluate the machine learning model’s performance. All statistical analysis was performed in Python (Version 2.7.3).

## Results

### Patients characteristics

Table 1 summarizes the patients’ baseline characteristics. Among 120 patients diagnosed with HCC, 88 (73.3%) were male. The underlying liver disease was distributed as follows, with HCV being the most prevalent cause (n = 61, 50.8). While non-alcoholic steatohepatitis (NASH) (n = 14, 11.7%), alcoholic liver disease (n = 11, 9.2%), HBV (n = 5, 4.2%), multiple etiologies (n = 14, 11.6%) and other pathologies (n = 15, 12.5%) were the other half. The majority of patients (n = 102, 85%) had one imaging-confirmed lesion at the time of diagnosis, followed by two (n = 15, 12.5%), and three (n = 3, 2.5%) lesions. The median (IQR) Meld-Na score without HCC exception points was 9 (5).

Among all patients, treatment modalities included surgical resection (n = 32, 26.7%), thermal ablation (n = 29, 24.2%), and OLT (n = 59, 49.1%). Disease recurrence was present in 45 (37.5%) patients and occurred more frequently in resected (n = 18, 56.3%) or ablated (n = 19, 65.5%) patients than in patients treated with OLT (n = 8, 13.6%). The mean time to recurrence was 26.8 months. Recurrent HCC appeared as intrahepatic local recurrence at the margin of the ablated/resected area (n = 10, 22.7%), intrahepatic distant recurrence remote from the original tumor site (n = 22, 50%), extrahepatic recurrence (n = 5, 11.4%) as well as at multiple locations (n = 7, 15.9%). The median (IQR) time from treatment to the first follow-up imaging was 166 days (326). There was no statistically significant difference in the clinical characteristics between recurrence and no-recurrence patients.

All MR images were re-examined according to the most recent LI-RADS criteria. In total, 93 patients (77.5%) were confirmed as being HCC-diagnosed with at least one LR-5 lesion on the pre-treatment MR imaging. The remaining 27 (22.5%) patients presented at most with one LR-4 (n = 17, 14.2%), LR-3 (n = 7, 5.8%), or LR-M (n = 3, 2.5%) lesion. Among these subjects, HCC was confirmed in 17 (14.2%) patients with liver biopsy and 10 (8.3%) patients with postoperative pathology. Table 2 summarizes the results of the LI-RADS reading.

**Table 2:** LI-RADS reading.

LI-RADS class, N (%)	Overall (n = 120)	Recurrence (n = 44)	No-recurrence (n = 76)
LR-5	93 (77.5)	38 (86.4)	55 (72.4)
LR-4	17 (14.2)	3 (6.8)	14 (18.4)
LR-3	7 (5.8)	3 (6.8)	4 (5.3)
LR-M	3 (2.5)	0 (0)	3 (3.9)

*LI-RADS* Liver Imaging Reporting and Data System.

### Prediction of recurrence

Table 3 depicts the predictive performance for all models. Six machine learning models were built to predict each patient’s recurrence probability within six different time frames. With the increasing length of the investigated time frame, the number of patients included in each model decreased, reducing the number of patients included from 120 in the 1-year model to 66 in the 6-year model.

**Table 3:** Performance metrics for each investigated time frame of recurrence.

	Time frame (years)						Total
	1	2	3	4	5	6	
N (recurrence)	120 (12)	116 (26)	98 (36)	82 (40)	74 (43)	66 (44)	120 (44)
Test-AUC mean (SD)	0.71	0.75	0.71	0.81	0.75	0.85	0.76 (0.05)

*AUC* area under the curve.

The test-AUC classification performance across all models ranged between (0.71–0.85). The variation in performance across all models was low, apparent by the standard deviation of their respective average test-AUC (AUC = 0.76, SD = 0.05). The test-AUC of each model is formed from one sample of predictions; hence standard deviations for these values are not provided.

### Recurrence-free survival

**Figure 2:**
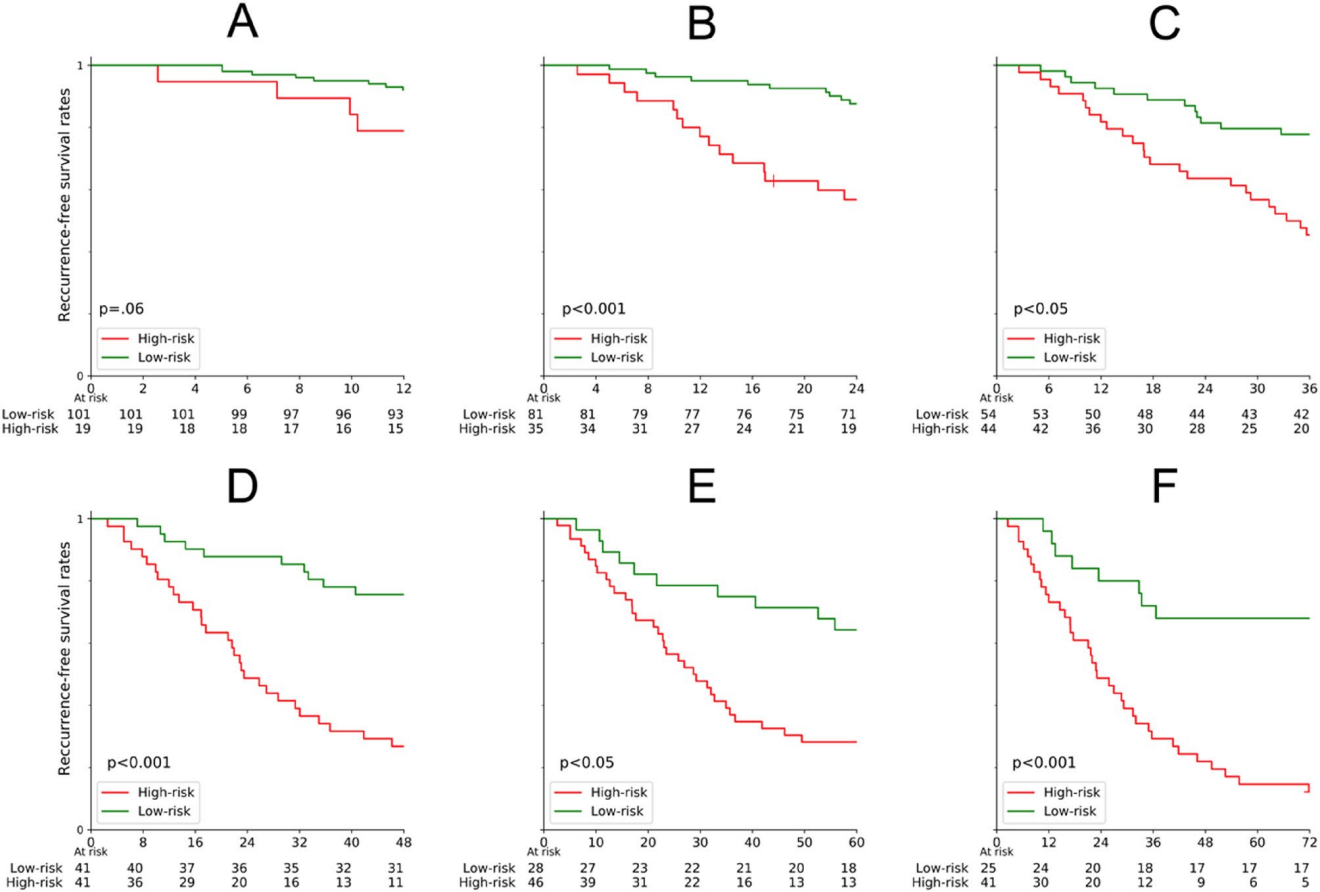
Kaplan–Meier analysis of RFS for each of the analyzed time frames according to algorithm predictions. The red curves represent patients the algorithm classifies as “recurrent” (High-risk), while the green curves represent patients the algorithm classifies as “non-recurrent” (Low-risk). Each event on the green curve represents prediction inaccuracy (false-negative) while each event on the red curve represents an accurate prediction of recurrence (positive predictive value).

Figure 2 shows the Kaplan–Meier plots for RFS. To investigate the algorithm’s clinical relevance the final recurrence predictions were analyzed with the Kaplan–Meier method. According to the model’s predictions, patients were stratified into “high-risk”- and “low-risk”-recurrence groups. The number of patients in each analysis corresponds to the number of patients in the corresponding recurrence prediction models, namely 120 (12), 116 (26), 98 (36), 82 (40), 74 (43), and 66 (44) for the 1–6 years models, respectively.

The analysis revealed a significant difference between predicted low- and high-risk patients in five of the six different recurrence time frames, namely for 2-, 3-, 4-, 5-, and 6-year recurrence time points. (log-rank test, p < 0.05). No statistical difference was obtained for the 1-year analysis (p = 0.06). A Kaplan–Meier analysis for each time frame and treatment can be found in Supplementary Fig. 3.

## Discussion

This study aimed to build, validate and test a deep learning approach to predict the post-treatment early-stage HCC recurrence risk from pre-treatment MR imaging. For all pre-defined time frames (ranging from 1 to 6 years), our model was able to predict tumor recurrence using baseline imaging data with moderate to high accuracy and test AUCs between 0.71 to 0.85. The subsequent Kaplan–Meier analysis showed that such predictions allow patients to be stratified into risk groups with significant differences in expected RFS at five recurrence time frames, with greater levels of certainty for the later time points. Overall, this study demonstrates that machine learning algorithms, specifically CNNs in combination with decision trees, can detect MR imaging features of HCC and background liver parenchyma associated with tumor recurrence in treated early-stage disease.

The relatively high prevalence of early disease recurrence following curative or potentially curative therapies of HCC continues to impede HCC, with 5-year recurrence rates being as high as 80%^3,26–28^. Among other unmet needs, current predominantly non-functional and lesion-size-based imaging criteria have proven to be of limited value when differentiating between patients with high-risk and low-risk for recurrence. There is scientific consensus that novel biomarkers for improved prediction of outcome are an unmet need with the hope to develop improved liver transplantation criteria that would incorporate factors beyond basic tumor morphology on cross-sectional imaging (number and size)^29^. Along those lines, our study was designed to apply currently available deep learning techniques to this unmet need. Our data demonstrated that this neural network generated reliable outcome prediction based on disease morphology on baseline imaging with a performance similar to or better than previously reported in related works^12,13,30–33^. While previous studies mainly used manually hand-crafted features based on human expert knowledge, our approach relies on automated feature extraction using a CNN. Overall, the clinical applicability of deep learning-based prediction of recurrences using baseline MR imaging will remain the subject of further study.

A direct comparison with prior works on HCC recurrence prediction is limited by the fact that most of the published studies use CT^34^ instead of MR imaging which is far less available for staging or screening purposes, particularly in Asia. Lv et al.^35^ proposed a deep-learning-based radiomics model to predict the 3-year recurrence of resected HCC from CT imaging, with a testing AUC of 0.83, notably higher than ours (0.71). Wang et al.^18^ presented a comparable study design to predict early HCC recurrence with an AUC of 0.825. Nevertheless, it remains difficult to deduct which modality is preferable given the use of different machine learning approaches, inclusion criteria, and number of patients.

A major technical advantage of the presented work is the use of the whole liver volume for algorithmic input rather than relying on segmented regions of the tumor or single slices. In comparison, Kim et al.^10^ attempted to predict early recurrences of HCC using quantitative imaging features, with the best results being obtained using a model that included the peritumoral environment (3 mm) rather than the tumor alone, impressively underlining the need to look beyond the malignancy itself. Additionally, Ji et al.^36^ demonstrated that in patients with cirrhosis, texture-specific imaging features of the liver background could be used to predict HCC recurrence, confirming that the underlying hepatic microenvironment as a whole may be reflective of the risk for recurrence*.* As HCC can be considered a secondary disease arising on top of a cirrhotic background, recurrent cancer may not only develop at the treated site but also de-novo. Therefore, whole liver input may overall be useful to account for underlying parenchymal conditions as an additional source of disease recurrence.

In the study, we chose the investigated recurrence period to be up to 6 years, as most post-operative HCC recurrences occur within the first 5–6 years. Our Kaplan–Meier analysis showed a statistically significant difference between low- and high-risk patients in five different time-to-recurrence (TTR) time frames with AUCs between 0.75 and 0.85. But our model was not statistically significant within 1 year, which is likely due to the small number of recurrent cases (n = 12) and the large data imbalance between recurrent and nonrecurrent cases (12 vs. 108).

Our study has some limitations. First, this is a retrospective single-center study with a relatively small cohort. The lack of external validation can be a source of bias and external algorithmic validation would be necessary for larger datasets before clinical deployment. Therefore, in light of the relatively small cohort and to prevent overfitting the data, we did not further pursue an independent test set at this point (e.g. 20% of the patients). However, the use of nested CV gives some confidence in the robustness of the learned models. As demonstrated in Table 1, we included nine HCC patients with end-stage liver cirrhosis (Child–Pugh-class C). According to former BCLC versions, these patients would haven been classified as terminal stage (BCLC D) disease patients and therefore no longer meet the inclusion criteria of our study. However, the most recent updates of the BCLC staging system^19^ allows for patients to be classified as early-stage and therefore be considered for transplant if their tumor burden meets transplant criteria. In our study, all nine patients were treated with OLT and therefore met the BCLC 2022 guidelines. In observance of the new BCLC 2022 algorithm, one patient in our cohort is formally staged as BCLC B but was downstaged to early-stage and ultimately met transplant criteria. Last, the LI-RADS reading was done as a split, with each of the radiologists reviewing a subset of the cases. A design where each radiologist reviewed all cases would have been preferable.

As we further our research and its application we intend to include various clinical features and modify the deep neural network to investigate whether there is an improvement in prediction accuracy. Some clinical parameters are already known to be associated with the recurrence of HCC, including gender, age, AFP^37^, NLR, or PLR^38^. We are hopeful that Ensemble methods that combine several predictors may potentially yield higher accuracy and add to the algorithm’s utility.

In conclusion, our work serves as proof of principle that deep learning-based algorithms can predict recurrence from pre-treatment MR imaging in patients with early-stage HCC. This study suggests that deep learning-based extraction of imaging features from baseline diagnostic imaging could be used to individualize treatment options with prognostications and adjust post-treatment follow-up in the sense of precision medicine. Future work should focus on enhancing the reliability of these algorithms with multi-center prospective cohort studies and the incorporation of clinical data.

## Supplementary Information

Supplementary Information.

## Data Availability

Data can be made available upon reasonable request to the corresponding author.
