# Supplementary material for: Predicting tumor recurrence on baseline MR imaging in patients with early-stage hepatocellular carcinoma using deep machine learning
Source: Sci Rep. 2023 May 10;13:7579. doi: 10.1038/s41598-023-34439-7 (PMC10172370; doi:10.1038/s41598-023-34439-7)
Supplement: Supplementary file 1 — Supplementary Information. [file 41598_2023_34439_MOESM1_ESM.pdf]

Supplementary Information

Title: Predicting tumor recurrence on baseline MR imaging in patients with early-stage hepatocellular carcinoma using deep machine learning

Journal: Scientific Reports

Authors:
Ahmet Said Kucukkaya, Tal Zeevi, Nathan Xianming Chai, Rajiv Raju, Stefan Philipp Haider, Mohamed Elbanan, Alexandra Petukhova-Greenstein, MingDe Lin, John Onofrey, Michal Nowak, Kirsten Cooper, Elizabeth Thomas, Jessica Santana, Bernhard Gebauer, David Mulligan, Lawrence Staib, Ramesh Batra, Julius Chapiro.

Correspondence
Julius Chapiro
Department of Radiology and Biomedical Imaging
Yale University School of Medicine
300 Cedar Street
New Haven, CT 06520
Tel: +1 203 785-2428
Email: julius.chapiro@yale.edu

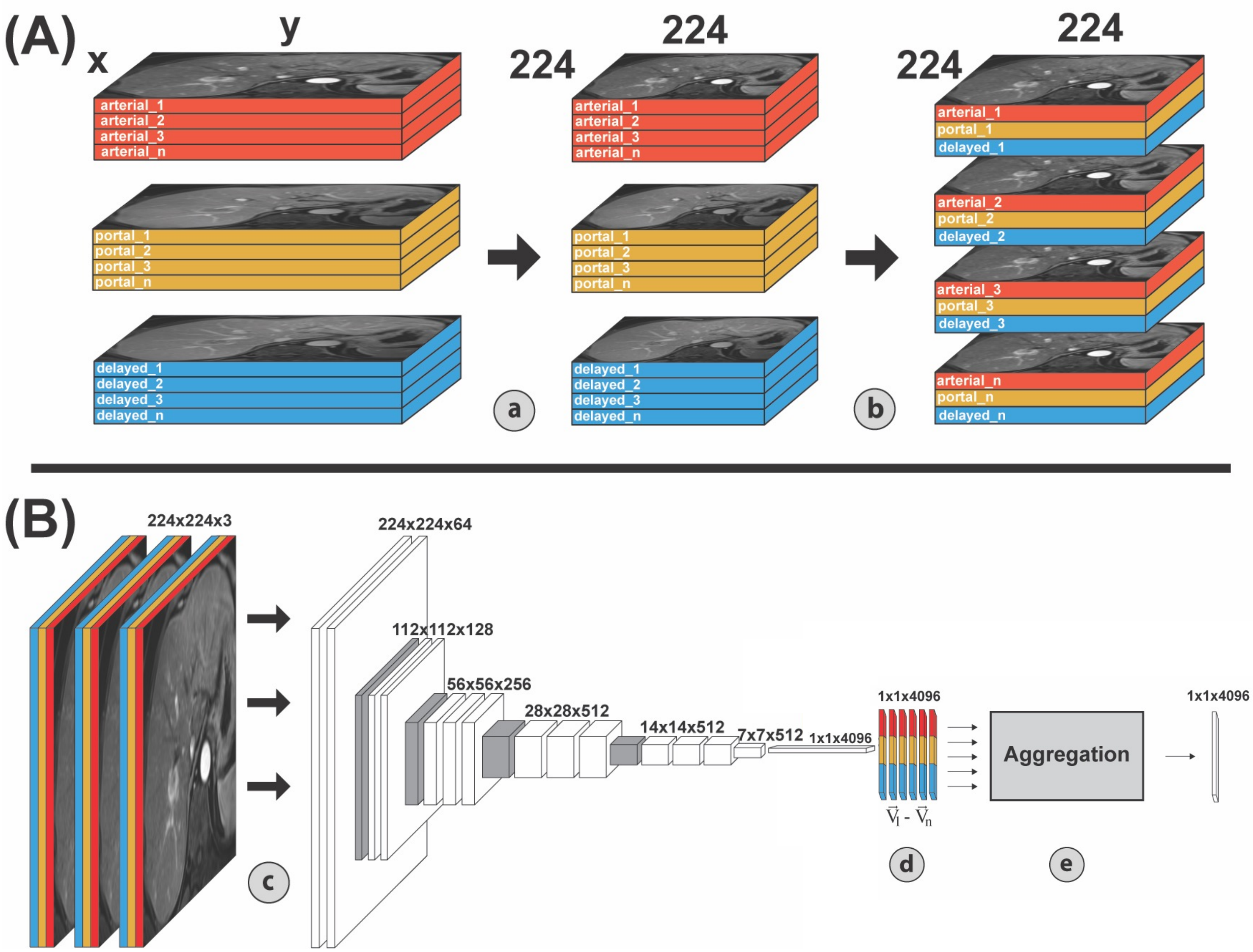

Supplementary Figure 1. Detailed overview of the (A) pre-processing and (B) feature extraction pipeline. (a) Tri-phasic MR images were resized to 224x224 pixels (b) and corresponding slices of each phase were stacked. The number of stacks for each patient equals the number of slices in the MR images. (c) Feature extraction was done using VGG16 (d) resulting in one feature vector per stack. (e) Finally, max pooling was applied to obtain a single feature value (aggregation) per patient.

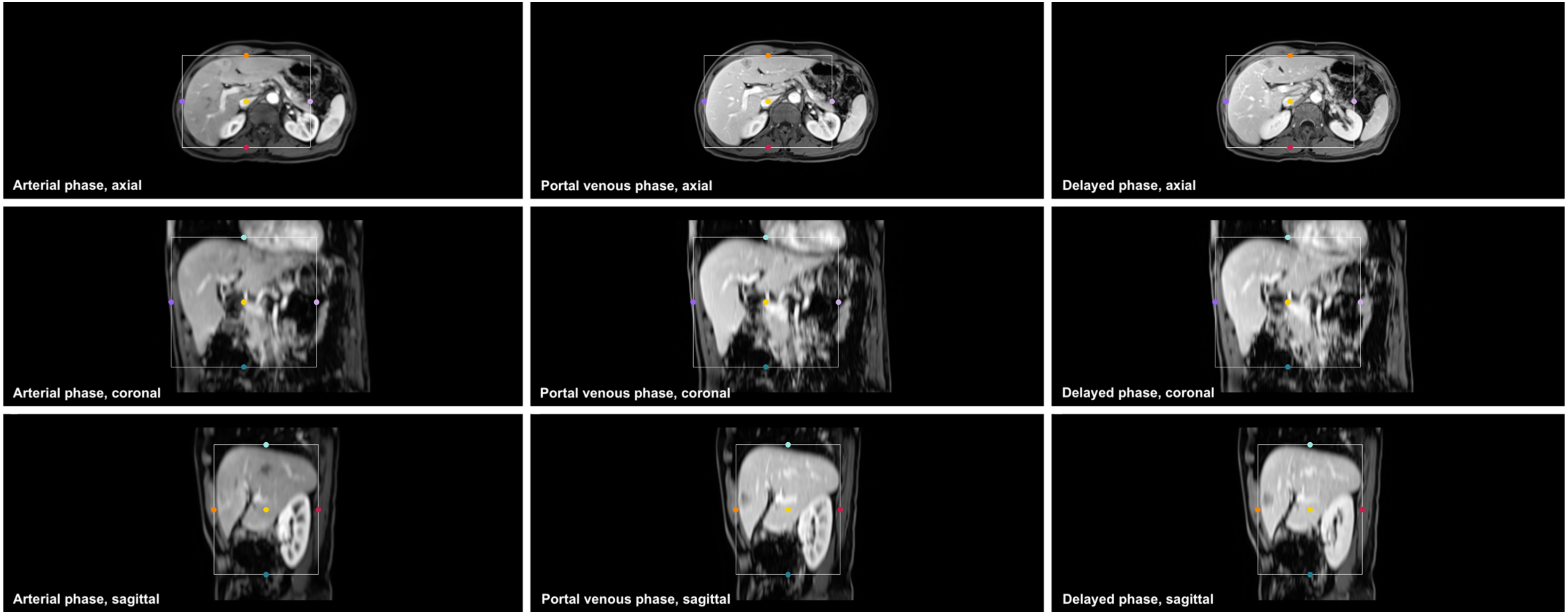

Supplementary Figure 2. Example of a bounding box, created in 3DSlicer. Each bounding box was drawn in arterial phase, and then adapted to portal venous and delayed phase imaging. The borders of the box are in line with the largest cross section in every dimension.

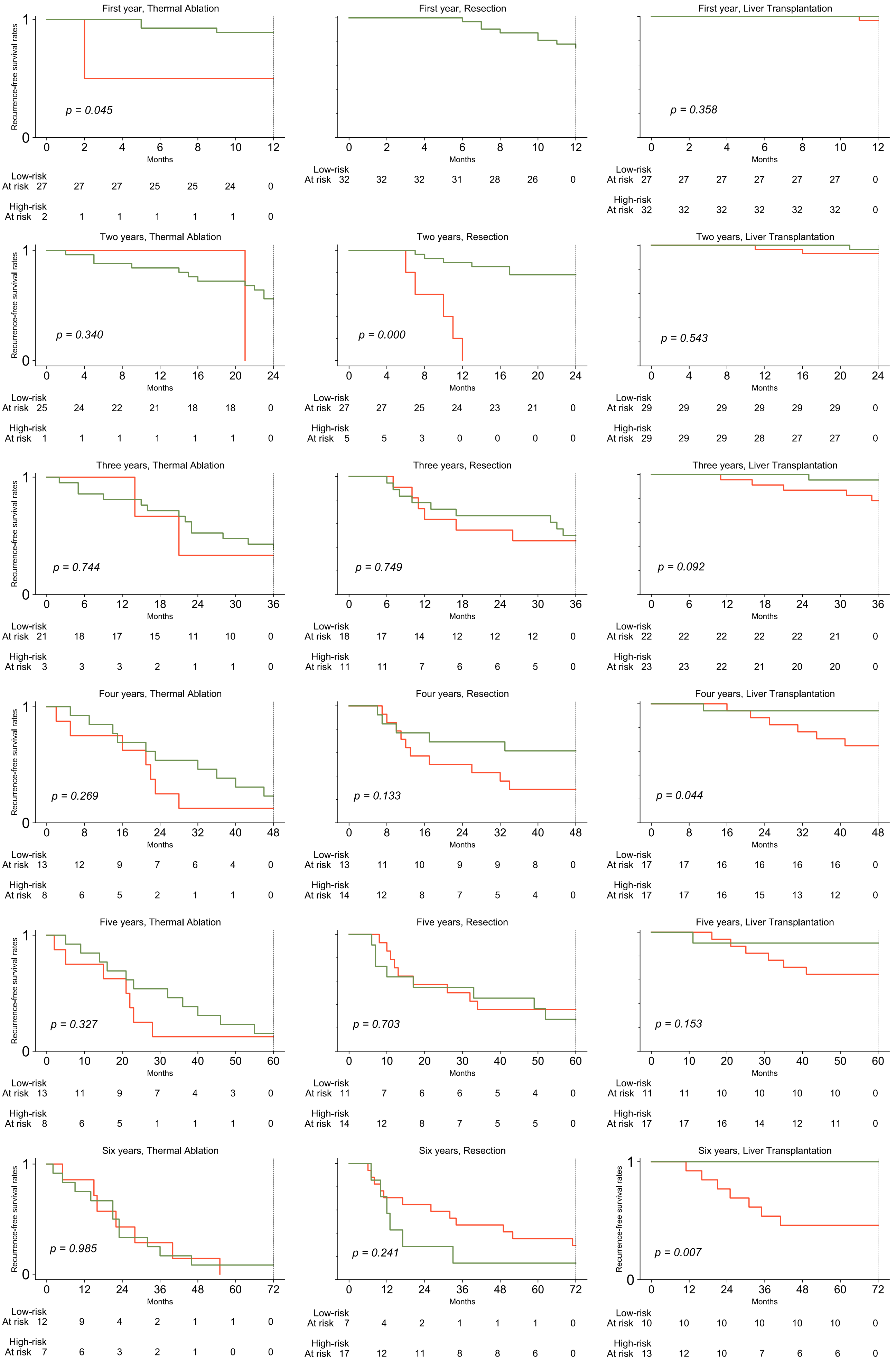

Supplementary Figure 3. Kaplan-Meier analysis of RFS for each analyzed time frame and treatment according to algorithm predictions. The plot "First year, Resection" shows only one curve because the algorithm classified all patients as low-risk recurrence.

Supplementary Table 1. Additional performance metrics for each time frame						
	**Time frame (years)**					
	**1**	**2**	**3**	**4**	**5**	**6**
**N (recurrence)**	120 (12)	116 (26)	98 (36)	82 (40)	74 (43)	66 (44)
**Accuracy**	0.81	0.75	0.67	0.74	0.69	0.8
**Sensitivity**	0.33	0.62	0.67	0.75	0.77	0.82
**Specificity**	0.86	0.79	0.68	0.74	0.58	0.77
**Positive Predictive Value**	0.21	0.46	0.55	0.73	0.72	0.88
**Negative Predictive Value**	0.92	0.88	0.78	0.76	0.64	0.68
**Matthews Correlation Coefficient**	0.16	0.37	0.33	0.49	0.35	0.57
